# Cost-Effectiveness of the Aerobika® Oscillating Positive Expiratory Pressure Device in the Management of Chronic Obstructive Pulmonary Disease Exacerbations in Canada

**DOI:** 10.1155/2019/9176504

**Published:** 2019-01-10

**Authors:** Nguyen Xuan Thanh, Philip Jacobs, Jason Suggett, Andrew McIvor, Alan Kaplan

**Affiliations:** ^1^Institute of Health Economics, 1200 10405 Jasper Avenue, Alberta, Canada; ^2^Department of Medicine, University of Alberta, Edmonton, Alberta, Canada; ^3^Trudell Medical International, London, Ontario, Canada; ^4^McMaster University, Hamilton, Ontario, Canada; ^5^Department of Family and Community Medicine, University of Toronto, Toronto, Ontario, Canada

## Abstract

**Background:**

The Aerobika® oscillating positive expiratory pressure (OPEP) device is a hand-held, drug-free medical device that has been shown to improve lung function and improve health-related quality of life in patients with chronic obstructive pulmonary disease (COPD). We estimated the cost-effectiveness of this device among postexacerbation COPD patients in the Canadian healthcare system.

**Methods:**

We performed a cost-utility analysis using a Markov model to compare both costs and outcome of patients with COPD who had recently experienced an exacerbation between 2 treatment arms: patients who used the Aerobika® device and patients who did not use the Aerobika® device. This cost-utility analysis included costs based on the Alberta healthcare system perspective as these represent Canadian experience. A one-year horizon with 12 monthly cycles was used.

**Results:**

For a patient after 1 year, the use of the Aerobika® device would save $694 in healthcare costs and produce 0.04 more in quality-adjusted life years (QALYs) in comparison with no positive expiratory pressure (PEP)/OPEP therapy. In other words, the economic outcome of the device was dominant (i.e., more effective and less costly). The probability for this device to be the dominant strategy was 72%. With a willingness to pay (WTP) threshold of $50,000 per QALY gained, the probability for the Aerobika® device to be cost-effective was 77%.

**Conclusions:**

Given one of the major treatment goals in the GOLD guidelines is to minimize the negative impact of exacerbations and prevent re-exacerbations, the Aerobika® OPEP device should be viewed as a potential component of a treatment strategy to improve symptom control and reduce the risk of re-exacerbations in patients with COPD.

## 1. Introduction

Chronic obstructive pulmonary disease (COPD) is a chronic disease, characterized by persistent respiratory symptoms and airflow limitation. The major cost factors of COPD are hospital admissions due to exacerbations [1]. Acute exacerbations are defined as periods of increased respiratory symptoms (e.g., dyspnea, cough, and mucus production) requiring treatment. A COPD exacerbation often leads to emergency room visits or hospital admissions. After an exacerbation, a patient can experience a reduction in functional ability and impaired quality of life. Of those patients hospitalized with COPD, approximately 20% will require rehospitalization within 30 days [2, 3].

The Global Strategy for the Diagnosis, Management, and Prevention of COPD and Global Initiative for Chronic Obstructive Lung Disease (GOLD) 2018 [4] guidelines propose that steps should be taken to minimize the impact of exacerbations and to prevent their reoccurrence. Symptomatic improvements can be sought through pharmacological and nonpharmacological therapies. Pharmacological treatments include the use of bronchodilators and inhaled corticosteroids, which are used concurrently with long-acting bronchodilators. These pharmacological treatments do not address the mucus buildup that contributes to airflow limitation [5]. Because of the mucus, there is a need to improve and maintain airway function in COPD patients, especially in the high-risk postexacerbation period when airways are most compromised.

Oscillating positive expiratory pressure (OPEP) devices are designed to expand the airways, mobilize mucus, and help to expel mucus from the lungs. The Aerobika® OPEP device (Trudell Medical International, Canada) is a hand-held, drug-free medical device that has been shown to perform these functions, thus improving lung function and health-related quality of life in patients with COPD [6]. A recent observational study using the Aerobika® device also demonstrated a significant decrease in the incidence of exacerbations leading to emergency room visits and hospitalizations among COPD patients [7]. In a US-based study, Khoudigian-Sinani et al. [8] previously estimated the cost-effectiveness of the Aerobika® OPEP device versus standard of care (i.e., no OPEP/positive expiratory pressure (PEP) therapy) among postexacerbation COPD patients. However, the US study did not consider the increase in mortality for severe exacerbations which may underestimate the effects of the device. Furthermore, differences in the healthcare system and costs between the US and Canada could limit the generalizability of the US study's results. Therefore, we used a similar model but accounted for the increase in mortality for severe exacerbations and Canadian costs, in order to estimate the cost-effectiveness of the same device in the Canadian healthcare system.

## 2. Materials and Methods

We performed a cost-utility analysis (CUA) [9] using a Markov model [10] to compare both the costs and outcome (quality-adjusted life years or QALYs) of patients with COPD who had recently experienced an exacerbation between 2 treatment arms: patients who used the Aerobika® device and patients who did not use the device (Aerobika® device was the unique difference between the 2 arms in terms of treatment). The cost-effectiveness analysis represented costs from the Alberta healthcare system perspective as these are well-documented costs for COPD in Canada. A one-year horizon with 12 monthly cycles was used to be comparable with the US study; [8] therefore, discounting for future costs and benefits was not applicable. The analysis was conducted using TreeAge Pro 2015 software (https://www.treeage.com/).

### 2.1. Model Structure

**Figure 1:**
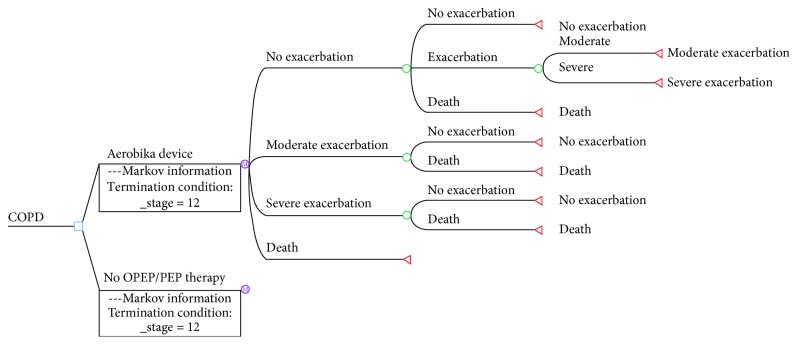
Model structure.

A patient with COPD enters the model in the “no-exacerbation” health state (Figure 1). Within a month (cycle), there are 3 possible outcomes for the patient: death, exacerbation (moderate or severe), or no exacerbation. If the patient experiences a moderate or severe exacerbation, within a month he/she could either die or revert to the no-exacerbation health state. A moderate exacerbation is defined as an emergency room (ER) visit by the patient, with no hospital admission. A severe exacerbation is defined as a patient's admission to hospital. [8] Mild exacerbation does not require any ER visit nor hospital admission, so it was treated as no exacerbation.

The structure is the same in both arms of the model. Conversely, transition probabilities are different between the arms of the model.

### 2.2. Model Inputs

Table 1 shows the model inputs used in this study. Based on an observational study of 30-day exacerbation outcomes in COPD patients by Burudpakdee et al. [7, 8] without treatment with any (oscillating or nonoscillating) PEP device, the probability of experiencing a moderate-to-severe exacerbation in the 1st month is 25.7% (Table 1). The probability in the 2nd to 12th months is 5.9% per month. In moderate-to-severe exacerbations, severe exacerbations account for 69.1% among patients who use the Aerobika® device and for 70.6% among no PEP/OPEP therapy patients. If using the Aerobika® device, the relative risk (RR) for moderate-to-severe exacerbations in the first month is 0.72 and for the 2nd to 12th months is conservatively assumed to be 1 (meaning no further improvement on exacerbations through using the Aerobika® device is assumed after the first month).

The probability of dying from a severe exacerbation is assumed to be 11%. This assumption is based on the results by Connors et al. [11, 12], where the authors studied 1,016 adult COPD patients who were hospitalized for exacerbations. They found that 11% of the patients died during their index hospital stays and 20% died within 60 days. The probability of dying among COPD patients was estimated at 0.01 per year. This assumption is based on previous studies which reported 12% of mild to moderate COPD patients died during 14 years of follow-up [11, 13], 4% of severe cases died during 26 months of follow-up [11, 14], and a severity distribution of COPD patients. We assumed that the severity distribution of COPD patients in Canada was similar to what reported by Hoogendoorn et al. (27% mild, 55% moderate, 15% severe, and 3% very severe) [15]. Conservatively, we assumed the probability of dying for patients who have had a moderate exacerbation was the same as the probability of dying among COPD patients (0.01 per year).

The costs for treatment of severe and moderate exacerbations were $13,119 and $456 per patient per month, respectively. These costs were estimated from Alberta Health Services (AHS) data. [16] Components of the cost for moderate exacerbations include ER costs and emergency doctor fees. Components of the cost for severe exacerbations include cost for the ER visit that precedes the hospital admission, hospital costs, emergency doctor fees, and attending respirologist fees. The cost for a severe exacerbation was based on the average length of stay in hospital which was estimated at 9.3 days [16]. Components of the cost for no exacerbation (outpatient follow-up) include physician visits and drugs (antibiotics and corticosteroids). The no-exacerbation cost was estimated at $65 per patient per month, by multiplying Alberta costs for physician visit ($78), antibiotics ($251), and corticosteroids ($101)^16^ with the utilization frequencies (0.131 physician visits, 0.172 antibiotic use and 0.113 corticosteroid use). Both doses of drugs and utilization of physician services were reported in Dhamane et al. [1, 8]. The cost of the Aerobika® device that was used was $90 per patient per year [17]. In our study, all costs were converted to 2017 Canadian dollars using the Bank of Canada general price index inflation calculator (https://www.bankofcanada.ca/rates/related/inflation-calculator/).

The utility score for people with COPD (0.897) was estimated by multiplying the utility score for mild (0.97), moderate (0.93), severe (0.72), and very severe (0.52) [18] with the severity distribution among COPD patients mentioned earlier [15]. Utility decrements for a month with severe (0.504) or moderate (0.12) exacerbations were estimated by multiplying the utility decrements per year (0.042 for severe and 0.01 for moderate exacerbation) [18] with 12 as used by Samyshkin et al. [19].

### 2.3. Scenario Analysis

We performed a scenario analysis where we assumed that the impact of the Aerobika® device on exacerbations (RR = 0.72) remained for the whole year [8].

### 2.4. Sensitivity Analysis

We performed both deterministic and probabilistic sensitivity analyses to account for the uncertainty of input parameters. In the deterministic sensitivity analysis, we used a one-way sensitivity analysis which allows one variable to vary at a time and reported variations in cost and in effect (QALY) in tornado diagrams. The range of each variable was assumed to be the base-case value (mean) ±20% as it was considered a reasonable range to evaluate a model parameter in the deterministic model [8]. In the probabilistic sensitivity analysis, which allows all variables to vary at a time, we ran 100,000 trials and reported probabilities for the Aerobika® device to be cost-effective. We assumed a beta distribution for probabilities and the utility of COPD people, a gamma distribution for cost and utility decrements, and a log-normal distribution for relative risks [20]. To estimate the standard deviation (SD), which is needed for TreeAge Pro 2015 software to calculate appropriate parameters for different types of distribution, we considered the ±20% range to be the 95% confidence interval which equates to mean ± 1.96 *∗* SD. For example, given the healthcare costs for severe exacerbation per patient per month is $13,119 (Table 1), the ±20% range would be $13,119 − $13,119 *∗* 20% = $10,495 to $13,119 + $13,119 *∗* 20% = $15,743. The SD was calculated as follows: given $13,119 ± 1.96 *∗* SD = $10,495 to $15,743, SD = ($13,119 − $10,495)/1.96 = $1339. Of note, the calculation is based on normal distribution parameters, and therefore, the results heavily rely on our parameterization choices.

## 3. Results

The base-case analysis results are shown in Table 2. For a patient after 1 year, the use of the Aerobika® device would save $694 in healthcare costs and produce 0.04 more in QALY outcomes in comparison with no PEP/OPEP therapy. In other words, the use of the Aerobika® device in the postexacerbation care population is dominant (i.e., more effective and less costly).

In the scenario analysis, a patient after using the Aerobika® device for 1 year would save $2,124 in healthcare costs and produce an additional 0.10 of a QALY, in comparison with no PEP/OPEP therapy (Table 3).

**Figure 2:**
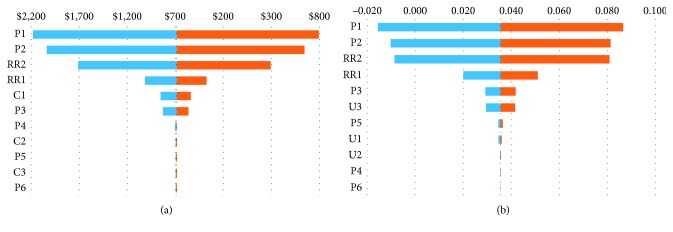
(a) Tornado diagram for incremental cost of Aerobika® OPEP device vs. no PEP/OPEP device. (b) Tornado diagram for incremental effect (QALY) of Aerobika® OPEP device vs. no PEP/OPEP device. *Note*. C1: cost per severe exacerbation, C2: cost per patient per month if no exacerbation, C3: cost per moderate exacerbation, P1: percentage of severe exacerbation if no Aerobika® device, P2: percentage of severe exacerbation if using Aerobika® device, P3: probability of exacerbation in the first month after exacerbation if no Aerobika® device, P4: probability of exacerbation between the 2nd and 12th months after exacerbation if no Aerobika® device, P5: probability of death among COPD patients with a severe exacerbation, P6: probability of death among patients with COPD (per year), RR1: relative risk of exacerbation in the first month between Aerobika® device and control groups, RR2: relative risk of exacerbation in the 2nd and 12th months between Aerobika® device and control groups, U1: utility score of patients with COPD (on average), U2: utility decrement for severe exacerbation (per month), U3: utility decrement for moderate exacerbation (per month).

Figures 2(a) and 2(b) show variations in incremental cost and effect using one-way sensitivity analyses. Accordingly, the largest range in incremental cost was from −$2,180 to +$795 (mean −$694), meaning that the use of the Aerobika® device would result in a range from $2,180 in reduced cost to an increase in $795. The largest range in the incremental outcome effect was from a reduction of 0.016 of a QALY to an increase of 0.087 (mean + 0.04). The most sensitive variable was the probability of a severe exacerbation in patients with no PEP/OPEP therapy. The second most sensitive variable was the probability of a severe exacerbation among patients using the Aerobika® device.

**Figure 3:**
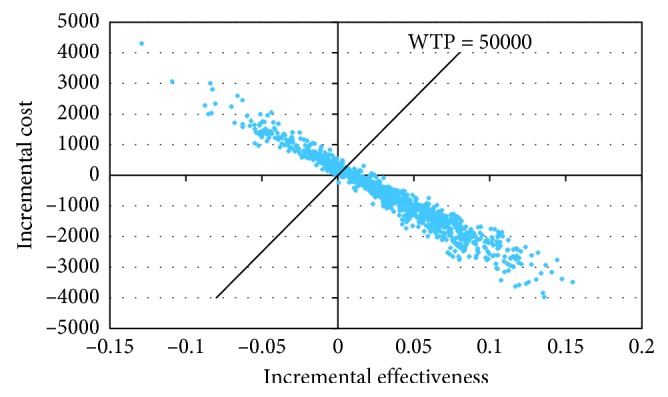
Incremental cost-effectiveness scatterplot.

The probabilistic sensitivity analyses (Figure 3: the cost-effectiveness plane) [21] show that the probability for the Aerobika® device to be the dominant strategy (both cost saving and more effective, the proportion of ICER in the quadrant VI of cost-effectiveness plane) was 72.2%. With a willingness to pay (WTP) threshold of $50,000 per QALY gained (the most commonly used WTP threshold in the Canadian literature), the probability for the Aerobika® device to be cost-effective was 76.8% (72.2% in the quadrant VI + 4.6% in the quadrant I and under the WTP line). The proportion of ICER in the quadrant I but above the WTP line was 2.3%. This proportion in the quadrant III was 0.1%. Of note, the probability for the Aerobika® device to be dominated (more costly and less effective, the proportion of ICER in the quadrant II of cost-effectiveness plane) was 20.8%. Due to the positive associations between the number of exacerbations and costs, and between the number of exacerbations and utility decrements, the Aerobika® device was (mostly) either dominant or dominated. That is, if the device prevented exacerbations then it was likely dominant because less exacerbations mean less costs and less utility decrements. On the other hand, if the device did not prevent exacerbations then it was likely dominated because more exacerbations mean more costs and more utility decrements.

## 4. Discussion

We conducted a model-based CUA which compared the Aerobika® OPEP device over a one-year period with standard care for persons with COPD. We used a government perspective to evaluate resource use and utility outcomes to allow comparability with other types of interventions. For both base-case and scenario sensitivity analyses, our results showed that, for a patient after 1 year, the use of the Aerobika® device would save $694 in healthcare costs and produce 0.04 more in QALY outcomes in comparison with no PEP/OPEP therapy. Using a willingness to pay for a QALY from $0 to $100,000 did not change the fact that the Aerobika® device would be dominant.

There is only one prior economic evaluation study [8] of this kind of device for persons with COPD. However, this study did not use utility as an outcome. The use of the utility variable allows us to generalize across interventions and policy scenarios.

Both our analysis and the US analysis by Khoudigian-Sinani et al. [8], were based on a real-world analysis of effectiveness [7] which used a thirty-day observation period for the use of the Aerobika® device. We believe that it is reasonable to assume that the effectiveness of the Aerobika® device would last for a year (the time horizon in our model) if its use continued; however, the extent of its protective benefits requires further investigation. Other limitations of our analysis should be mentioned. There is limited published evidence on the evaluation of OPEP devices to benchmark against. We used evidence of effectiveness from a hospitalized sample; results for a nonhospitalized sample were not available.

Data from the effectiveness study on which our model is based come from a US study. While the effectiveness is based on US data, the estimate of hospital readmission may differ from that in Canada. In the US, hospitals are penalized for patient readmission within a 30-day period; there is therefore an incentive for hospitals to prevent patients from being readmitted. There is no such incentive in Canada, where rehospitalisation rates may be greater. We did not have any corroborative evidence for Canada, but we did allow for this difference in our sensitivity analysis. Due to lack of data, the variation of parameters in sensitivity analyses was assumed to be ±20% from the base-case value (mean) which may not exactly be the 95% CI of parameters. Therefore, more research is needed to confirm the cost-effectiveness of Aerobika® device.

## 5. Conclusion

Given one of the major treatment goals in the GOLD guidelines is to minimize the negative impact of exacerbations and prevent re-exacerbations, the Aerobika^∗^ OPEP device should be viewed as a potential component of a treatment strategy to improve symptom control and reduce risk of re-exacerbations in patients with COPD.

## Figures and Tables

**Table 1:** Model inputs.

Model inputs	Mean	Low	High	Data source
*Monthly event probabilities in the study population*				
Probability of exacerbation in the 1st month	0.257	0.206	0.308	[7, 8]
Probability of exacerbation after the 1st month	0.059	0.047	0.071	[7, 8]
Probability of dying for severe exacerbation	0.110	0.088	0.132	[9, 10]
Probability of dying for no or moderate exacerbation^*∗*^	0.010	0.008	0.012	[9, 11–13]
*Aerobika® device effect on exacerbations*				
Relative risk of exacerbation in the 1st month	0.720	0.576	0.864	[7, 8]
Relative risk of exacerbation after the 1st month	1.000	0.800	1.200	[8]
*Distribution of exacerbations*				
Severe exacerbation if using Aerobika® device	0.691	0.553	0.830	[8]
Severe exacerbation if not using Aerobika® device	0.706	0.565	0.848	[8]
*Healthcare cost per patient per month*				
Severe exacerbation	$13,119	$10,495	$15,743	[14]
Moderate exacerbation	$456	$365	$547	[14]
No exacerbation	$65	$52	$78	[1, 8, 14]
*Cost of Aerobika® device per patient per year*	$90			[15]
*QALY*				
Utility score for people with COPD	0.897	0.718	1.000	[13, 16]
Utility decrement if severe exacerbation (per month)	0.504	0.403	0.605	[16, 17]
Utility decrement if moderate exacerbation (per month)	0.120	0.096	0.144	[16, 17]

^*∗*^Per year.

**Table 2:** Base-case analysis results: incremental cost and incremental effect.

	Aerobika® device	No PEP/OPEP therapy
Total direct medical cost	$8,141.56	$8,835.71
Total QALYs	0.57	0.53
Incremental cost^*∗*^	−$694.15	
Incremental effect (QALY gained)^*∗*^	0.04	
Incremental cost-effectiveness ratio (ICER)	Aerobika® device is the dominant strategy	

^*∗*^For variations, please see sensitivity analyses.

**Table 3:** Scenario analysis results: incremental cost and incremental effect.

	Aerobika® device	No PEP/OPEP therapy
Total direct medical cost	$6,712.19	$8,835.71
Total QALYs	0.63	0.53
Cost savings	$2,123.52	
QALY gained	0.1	
Incremental cost-effectiveness ratio (ICER)	Aerobika® device is the dominant strategy	

## Data Availability

The transition probabilities, utility scores, and costs used to support the findings of this study are included within the article.

## Abbreviation List

AHS: Alberta Health Services
COPD: Chronic obstructive pulmonary disease
CUA: Cost-utility analysis
ER: Emergency room
GOLD: Global Initiative for Chronic Obstructive Lung Disease
OPEP: Oscillating positive expiratory pressure
PEP: Positive expiratory pressure
QALY: Quality-adjusted life year
RR: Relative risk
SD: Standard deviation
US: United States
WTP: Willingness to pay.

## References

[1] Dhamane A., Moretz C., Zhou Y. (2015). COPD exacerbation frequency and its association with health care resource utilization and costs. *International Journal of Chronic Obstructive Pulmonary Disease*.

[2] Shah T., Press V. G., Huisingh-Scheetz M., White S. R. (2016). COPD readmissions. *Chest*.

[3] Jencks S. F., Williams M. V., Coleman E. A. (2009). Rehospitalizations among patients in the medicare fee-for-service program. *New England Journal of Medicine*.

[4] (2018). *Global Strategy for the Diagnosis, Management and Prevention of COPD, Global Initiative for Chronic Obstructive Lung Disease (GOLD)*.

[5] Burgel P. R., Martin C. (2010). Mucus hypersecretion in COPD: should we only rely on symptoms?. *European Respiratory Review*.

[6] Svenningsen S., Paulin G. A., Sheikh K. (2016). Oscillatory positive expiratory pressure in chronic obstructive pulmonary disease. *COPD: Journal of Chronic Obstructive Pulmonary Disease*.

[7] Burudpakdee C., Seetasith A., Dunne P. (2017). A real-world study of 30-day exacerbation outcomes in chronic obstructive pulmonary disease (COPD) patients managed with Aerobika OPEP. *Pulmonary Therapy*.

[8] Khoudigian-Sinani S., Kowal S., Suggett J. A., Coppolo D. P. (2017). Cost-effectiveness of the Aerobika^∗^ oscillating positive expiratory pressure device in the management of COPD exacerbations. *International Journal of COPD*.

[9] Drummond M. F., Schulper M. J., Claxton K., Stoddart G. L., Torrance G. W. (2015). *Methods for the Economic Evaluation of Health Care Programmes*.

[10] Sonnenberg F. A., Beck J. R. (1993). Markov models in medical decision making: a practical guide. *Medical Decision Making*.

[11] Celli B. R. (2010). Predictors of mortality in COPD. *Respiratory Medicine*.

[12] Connors A. F., Dawson N. V., Thomas C. (1996). Outcomes following acute exacerbation of severe chronic obstructive lung disease. The SUPPORT investigators (study to understand prognoses and preferences for outcomes and risks of treatment). *American Journal of Respiratory and Critical Care Medicine*.

[13] Anthonisen N. R., Skeans M. A., Wise R. A., Manfreda J., Kanner R. E., Connett J. E. (2005). The effects of a smoking cessation intervention on 14.5-year mortality: a randomized clinical trial. *Ann Intern Med*.

[14] Sin D. D., Wu L., Anderson J. A. (2005). Inhaled corticosteroids and mortality in chronic obstructive pulmonary disease. *Thorax*.

[15] Hoogendoorn M., Feenstra T. L., Schermer T. R., Hesselink A. E., Rutten-van Mölken M. P. (2006). Severity distribution of chronic obstructive pulmonary disease (COPD) in Dutch general practice. *Respiratory Medicine*.

[16] Alberta Health (April 2018). Interactive health data application. http://www.ahw.gov.ab.ca/IHDA_Retrieval/selectCategory.do?dataBean.id=201&command=doSelectSubCategory&cid=201.

[17] *Data Obtained from Trudell Medical International*.

[18] Rutten-van Mölken M. P., Hoogendoorn M., Lamers L. M. (2009). Holistic preferences for 1-year health profiles describing fluctuations in health: the case of chronic obstructive pulmonary disease. *Pharmacoeconomics*.

[19] Samyshkin Y., Schlunegger M., Haefliger S., Ledderhose S., Radford M. (2013). Cost-effectiveness of roflumilast in combination with bronchodilator therapies in patients with severe and very severe COPD in Switzerland. *International Journal of Chronic Obstructive Pulmonary Disease*.

[20] Briggs A., Claxton K., Sculpher M. (2006). *Decision Modelling for Health Economic Evaluation*.

[21] Black W. C. (1990). The CE plane: a graphic representation of cost-effectiveness. *Medical Decision Making*.

