# Corrigendum to “Cost-Effectiveness of the Aerobika® Oscillating Positive Expiratory Pressure Device in the Management of Chronic Obstructive Pulmonary Disease Exacerbations in Canada”

**DOI:** 10.1155/2019/5906984

**Published:** 2019-05-19

**Authors:** Nguyen Xuan Thanh, Philip Jacobs, Jason Suggett, Andrew McIvor, Alan Kaplan

**Affiliations:** ^1^School of Public Health, University of Alberta, Edmonton, Alberta, Canada; ^2^Department of Medicine, University of Alberta, Edmonton, Alberta, Canada; ^3^Trudell Medical International, London, Ontario, Canada; ^4^McMaster University, Hamilton, Ontario, Canada; ^5^Department of Family and Community Medicine, University of Toronto, Toronto, Ontario, Canada

In the article titled, “Cost-Effectiveness of the Aerobika® Oscillating Positive Expiratory Pressure Device in the Management of Chronic Obstructive Pulmonary Disease Exacerbations in Canada” [1], the affiliation of the first author was incorrect. Also, the e-mail address of the first/corresponding author has been updated. The corrected authors' list and affiliations are shown above.
